# Supplementary material for: Regulation of posterior body and epidermal morphogenesis in zebrafish by localized Yap1 and Wwtr1
Source: eLife. 2017 Dec 28;6:e31065. doi: 10.7554/eLife.31065 (PMC5773182; doi:10.7554/eLife.31065)
Supplement: Supplemental file 3. [file elife-31065-fig3.docx]

**Supplemental Table 3: primers used to make in situ hybridization probes**

| **ENSEMBL gene name** | **Fwd primer** | **Rev primer** |
| --- | --- | --- |
| *ecrg4b* | ggcggcgaattcATAATGTCTCTCCACAGCCTCTGTG | ggcggcaagcttATTTTAGCCAGAATCCTGCAGCTC |
| *CABZ01115881.1* | ggcggcggatccATCATGAACAATCACTCGATCTCGC | ggcggcgaattcCGGAGGTTACTGTCAGATTGTGC |
| *amotl2b* | ggcggcggatccAAAATGAGAGGTGAAGAAGCATCCG | ggcggcctcgagCTGCTCTTCATTCTGCAGATGGAG |
| *zgc:110712* | ggcggcggatccCAGAAGTTCAGCTCTCAGAGTGTG | ggcggcctcgagTGGAGCTGACTCAGCTGGG |
| *wu:fc23c09* | ggcggcggatccTAGCATTCAGTTAAACTTGCTCTCCC | ggcggcgaattcCCACTTGAAAGCCTCTGAGGC |

The restriction sites used for cloning are underlined.
