# Supplementary material for: Regulation of posterior body and epidermal morphogenesis in zebrafish by localized Yap1 and Wwtr1
Source: eLife. 2017 Dec 28;6:e31065. doi: 10.7554/eLife.31065 (PMC5773182; doi:10.7554/eLife.31065)
Supplement: Supplemental file 4. [file elife-31065-fig4.docx]

**Supplemental Table 4: primers for qPCR**

| **Gene** | **Fwd primer** | **Rev primer** |
| --- | --- | --- |
| *rpl13* | TAAGGACGGAGTGAACAACCA | CTTACGTCTGCGGATCTTTCTG |
| *cyr61* | ACGCGAAGATGTTTGCTTGG | CCGGCCCTCTGATTTAGCTC |
| *ctgfa* | GTGTTCACCTGGTGTAAGCCTAGTTC | ACCGTCCAGACACGTGCACTGGTAT |
| *dcn* | TACCTTTCTGCCCCTTCCGA | GGAGACCCTTCAGGCCTTTG |
| *cdc42ep3* | AGGGACGGTTCACTCACAAAA | GACCTTCTGGAGTCTTGCAGT |
| *csrp1a* | GAAGTGCAATGTGAAGGGCG | GTAGCCGTAGCCTTTGGGTC |
| *col1a1b* | GCTGGTGCTACTGGACCTAAA | GGGCTCCTCTTTGTCCCTCT |
| *pcdh7b* | GTGTCGGTGAGGCAGAGCATA | GGAGGTCATGAAGGTGGAGAGTTT |
| *ecgr4b* | TCCAGGGTGGCGTTGTTCATC | TCTCGTTTGATGAGGATCCTGTG |
| *CABZ01115881.1* | TGCAGGTCAACACCAACAAC | CTGTGCGGAGGTTACTGTCA |
